# Development of Paroxetine Hydrochloride Single Layer Controlled-Release Tablets Based on 3^2^ Factorial Design

**DOI:** 10.3390/pharmaceutics10040243

**Published:** 2018-11-20

**Authors:** Yao Yang, Zhengwei Huang, Xuan Zhang, Jinyuan Li, Ying Huang, Wanxin Chen, Xin Pan, Chuanbin Wu

**Affiliations:** 1 School of Pharmaceutical Sciences, Sun Yat-Sen University, Guangzhou 510006, China; yangyao5@mail2.sysu.edu.cn (Y.Y.); hzhengw3@mail2.sysu.edu.cn (Z.H.); zhangx57@mail2.sysu.edu.cn (X.Z.); chenwx49@mail2.sysu.edu.cn (W.C.); wuchuanb@mail.sysu.edu.cn (C.W.); 2 State Key Laboratory of Oncology in South China, Collaborative Innovation Center for Cancer Medicine, Sun Yat-Sen University Cancer Center, Guangzhou 510060, China; lijiny@sysucc.org.cn

**Keywords:** depressive disorder, paroxetine hydrochloride, factorial design, single layer controlled-release tablet, PAXIL^®^ CR, oral bioavailability

## Abstract

Major depressive disorder (MDD) is one of the main contributors to disability and suicide mortality globally. Paroxetine hydrochloride (PHH) is the most potent antidepressant used for MDD treatment. Due to its reduced side effects PAXIL^®^ CR is a widely-used controlled-release formulation of PHH. However, the complicated double-layer production of PAXIL^®^ CR faces the risk of layer separation. In this study, PHH enteric coating single layer controlled-release tablets (PHH-EC-SLTs) were designed as a simplified substitution of PAXIL^®^ CR through a rational formulation screening. The optimized PHH-EC-SLTs showed similar release behaviors in vitro to PAXIL^®^ CR and the release profiles corresponded to a zero-order release model (*R*^2^ = 0.9958). Polymer matrix erosion was the main release mechanism, according to the fitting exponents *n* > 1 in the Korsmeyer-Pappas model. Crucial pharmacokinetic parameters including peak-reaching time (*T*_max_), peak concentration (*C*_max_) and the area under the blood level-time curve (AUC_0-48_) of PHH-EC-SLTs and PAXIL^®^ CR had no significant difference (*p* > 0.05) and the relative bioavailability (*F* = 97.97%) of PHH-EC-SLTs demonstrated their similar pharmacokinetic profiles in vivo. In view of avoiding layer separation risk and simplifying the preparation processing, the self-made PHH-EC-SLTs could be considered as a safe and economic alternative to PAXIL^®^ CR.

## 1. Introduction

Major depressive disorders (MDDs) are common mental disorders diagnosed in nearly all age groups and all world regions [1]. Globally, the total number of patients with depression increased by 18.4% between 2005 and 2015 [2], and was estimated to exceed 300 million in 2015 [3]. MDD was ranked by WHO as one of the largest contributors to global disability (7.5% in 2015). It was also one of the major contributors to suicide deaths, whose number was close to 800,000 per year [3].

Much effort has been made to develop an effective therapy for MDD. Antidepressant administration was considered as an important treatment [4]. Paroxetine hydrochloride (PHH) is the most potent antidepressant approved for the treatment of MDD [5]. It has the highest affinity towards the serotonin reuptake inhibitors with a binding affinity of 0.10 nmol/L, and virtually no affinity for other receptors like histaminic, α- or β-adrenoceptor, dopaminergic or serotonergic receptors [6,7], which means a mild side effect.

Administration of conventional PHH tablets may induce nausea and lead to early treatment discontinuation due to the gastrointestinal reactions and the remarkable peak-valley fluctuation of its plasma concentration. PAXIL^®^ CR is a controlled-release tablet produced by GlaxoSmithKline which adopted the patented Geomatrix^®^ technology of Skypepharma. It can significantly reduce the abovementioned side effects [8,9], which is beneficial for its steady drug release behavior [10]. Thus, PAXIL^®^ CR finds wider application among the PHH formulations.

**Figure 1:**
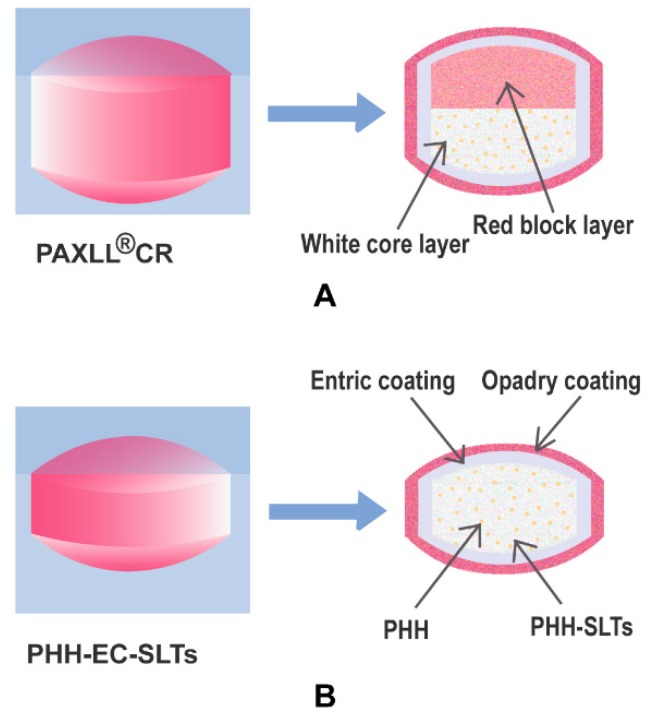
Schemes of PAXIL^®^ CR (**A**) and PHH-EC-SLTs (**B**), shown in longitudinal sections.

Geomatrix^®^ is an oral drug delivery system technology characterized by multilayer hydrogel matrix tablets, it is applicable to either poorly or highly soluble drugs and various drug release profiles can be obtained, including multiphase and zero-order release [11]. Specifically, PAXIL^®^ CR has a red block layer without drug and a white core layer with PHH as shown in Figure 1A. The main function of the red block layer is to maintain the steady drug release [12,13,14] of PAXIL^®^ CR from the white core layer, which shows zero-order release. The red block layer could decrease the contact between the drug and dissolution medium in the early stage to reduce the initial burst release. As time goes by, the red block layer is corroded gradually and the white core layer with PHH is swollen by water, so the surface area between PHH and dissolution medium is increased which would accelerate the PHH release in the later stage. Despite the advantages of PAXIL^®^ CR, its preparation procedure is rather complicated, and therefore the risks of preparation failure and double-layer separation may occur. Considering the important function of the red block layer, the drug release behavior of PAXIL^®^ CR would change and it would not achieve the desired zero-order release. The PHH release would also fail to be controlled if the two layers stick together insufficiently. These changes in release behavior may result in unknown and potentially dangerous side effects. Besides, the complicated preparation method also increases the production costs.

To solve the abovementioned problems, in this work PHH enteric coating single layer controlled-release tablets (PHH-EC-SLTs) were designed as an alternative of PAXIL^®^ CR. That was because in the PHH-EC-SLTs, the drug release control was based on the properties of the composite gel skeleton (CGS) in the only layer of tablets without another helping layer. Previous studies showed that the CGS of hydroxypropylmethyl cellulose (HPMC) and sodium carboxymethyl cellulose (CMC-Na) or hydroxypropyl cellulose (HPC) and CMC-Na had a more stable sustained-release profile compared to the individual polymers [15]. We took this concept one step further by preparing propranolol hydrochloride and tramadol hydrochloride skeleton tablets by adjusting the proportion of xanthan gum, guar gum and HPMC, respectively. Both two gel skeleton tablets possessed zero-order release in vitro [16,17]. This was based on the fact that the hydration of HPMC K4M was much slower than that of other polymers like CMC-Na, but the gel strength of HPMC K4M was higher than that of CMC-Na. As a result, the combined application of HPMC K4M and CMC-Na would adjust satisfactory hydration and strength of these two materials and achieved an ideal sustained release effect. Therefore, CGS might be a promising choice for the development of the simple and safe PHH-EC-SLTs with zero-order release patterns. Moreover, the risks of rupture of bilayers would be avoided and the preparation process would be significantly simplified, since the PHH-EC-SLTs prepared from CGS could exhibit sustained-release without the help of a red block layer. It is believed that the development of PHH-EC-SLTs can bring economic and social benefits.

HPMC K4M and CMC-Na are water absorbing or swelling polymers which are commonly used in the preparation of sustained release tablets. In this work, based on the related literature and our previous study, HPMC K4M and CMC-Na were chosen as to formulate the CGS system. An accurate selection of the excipients is very important for the development of drug delivery systems [18]. In order to obtain qualified PHH-EC-SLTs, a rational formulation development should be performed. Firstly, a 3^2^ factorial design [19,20,21] was applied for the optimization of CGS system for PHH-SLTs. Then, single factor experiments were used to optimize other formulation and processing parameters. PHH-SLTs were further coated with acrylic resin to produce PHH-EC-SLTs. A scheme of PHH-EC-SLTs is shown in Figure 1B.

The good product reproducibility of PHH-EC-SLTs suggests that the preparation technology is practical and repeatable. It was demonstrated that the self-made PHH-EC-SLTs with a simple preparation process had similar release behaviors in vitro and pharmacokinetic profiles in vivo compared to PAXIL^®^ CR. Thus, the optimized PHH-EC-SLTs formulation developed in this work may be a prospective alternative to PAXIL^®^ CR and will be beneficial for advancing the current therapy of MDD.

## 2. Materials and Methods 

### 2.1. Materials and Animals

PHH was purchased from Zhejiang Linhai Jinqiao Chemical Co., Ltd. (Zhejiang, China). HPMC K4M was obtained from Colorcon Co., Ltd. (Shanghai, China). CMC-Na was provided by Guangzhou Qihua Medical Equipment Co., Ltd. (Guangdong, China). Polyvinylpyrrolidone (PVP K30) was purchased from International Specialty Products Inc. (Wayne, NJ, USA). Microcrystalline cellulose (MCC) and magnesium stearate were purchased from Huzhou Pharmaceuticals Co., Ltd. (Zhejiang, China). Lactose (Granulac 200) was obtained from Molkerei Meggle Wasserburg GmbH & Co. Kg. (Wasserburg, Germany). Methacrylic resin (Eudragit L30D-55) was received from Evonik Röhm GmbH (Darmstadt, Germany). The reference substance of PHH, desfluoroparoxetine and *N*-methyl-paroxetine were supplied by the National Institutes for Food and Drug Control (Beijing, China). PAXIL^®^ CR was purchased from GlaxoSmithKline plc. (Brentford, UK) and applied as the reference formulation. Clozapine was obtained from Shandong Qilu Pharmaceuticals Co., Ltd. (Shandong, China). Methanol, acetonitrile and *n*-hexane were of chromatographic grade. All other chemicals used were of analytical grade. Beagle dogs (male, 4-year old) were purchased from Kangda Experimental Animal Technology Co., Ltd. (Guangdong, China). Dogs were maintained in separate cages and food and water were provided ad libitum.

### 2.2. Development of PHH-EC-SLTs

PHH-SLTs were prepared with a CGS system composed of HPMC K4M and CMC-Na. The CGS system was optimized with a 3^2^ factorial design, and other formulation and processing parameters of PHH-SLTs were determined by single factor experiments. Then, PHH-SLTs were coated to obtain PHH-EC-SLTs.

#### 2.2.1. Factorial Design 

A 3^2^ factorial design was applied to optimize the content of the two critical excipients (HPMC K4M and CMC-Na). Their individual or combined effects on the formulation were evaluated. The compositions for the factorial design were summarized in Table 1, lactose was added to build up the 100% content.

Mean dissolution time (MDT) is the sum of different periods of time during which drug molecules or the dose stay in the polymer before release, divided by the total number of molecules or the total dose, respectively [22]. It could be used to describe the drug release rate in vitro. MDT was calculated by Equation (1):

(1) $$\mathrm{MDT}=\frac{\sum_{i=1}^{i=n}t_{\mathrm{mid}}\times △M}{\sum_{i=1}^{i=n}△M}$$

where *i* was the number of drug release sampling, *n* was the total number of drug release sampling, *t*_mid_ was the median time from *i*−1 to *i*, and ∆*M* was the increase of drug release from *i*−1 to *i*.

The dissolution curve of each formulation was compared with that of the reference formulation. Similarity factor (*f*_2_) was employed to investigate the similarity of different dissolution curves, and *f*_2_ was calculated according to Equation (2):

(2) $$f_{2}=50\times \log\left\{{\left[1+\left(\frac{1}{n}\right)\sum_{t=1}^{n}{\left(R_{t}-T_{t}\right)}^{2}\right]}^{-0.5}\times 100\right\}$$

where *R*_t_ was the drug release rate of reference substance at time *t*, *T*_t_ was the release rate of specimen at time *t*, and *n* was the total number of drug release sampling. A high *f*_2_ value meant little differences between two formulations. In general, it was considered that two formulations have similar release behaviors in vitro if *f*_2_ ≥ 50.

Statistical data was subjected to variance analysis (one-way ANOVA) and further binominal regression fitting with Equation (3) (*Y* was the dependent variable, *X*_1_ and *X*_2_ were independent variable and the other parameters were constant) by SPSS 22.0 (IBM Corp., Armonk, NY, USA). They were considered significant when *p* < 0.01. Statistical results were obtained using Sigma Plot 13.0 (Systat Software, Inc., San Jose, CA, USA) to obtain three-dimensional response surface of *f*_2_ or MDT as a function of the amount of HPMC and CMC-Na:

(3) $$Y=\mathrm{Intercept}+b_{1}X_{1}+b_{2}X_{2}+b_{11}X_{1}X_{1}+b_{22}X_{2}X_{2}+b_{1}b_{2}X_{1}X_{2}$$

#### 2.2.2. Single Factor Experiments 

The effects of different types of fillers, content of adhesives, tableting method, tableting pressure and solvent on the drug release behavior were investigated by single factor experiments. In brief, PHH, HPMC K4M, CMC-Na and other excipients were sieved through 200# mesh (75 μm). Then, the sieved drug and excipients were precisely weighed and mixed homogeneously with appropriate volume of ethanol-water solution and then granulated by passing through 20# mesh (0.9 mm). Next, humid granules were dried in an oven at 40 °C, and then passed through 18# mesh (1.0 mm) to obtain uniform dry granules. Finally, the dry granules were mixed with magnesium stearate and silicon dioxide for the compressing procedure to prepare tablets.

#### 2.2.3. Coating for PHH-SLTs

**Figure 2:**
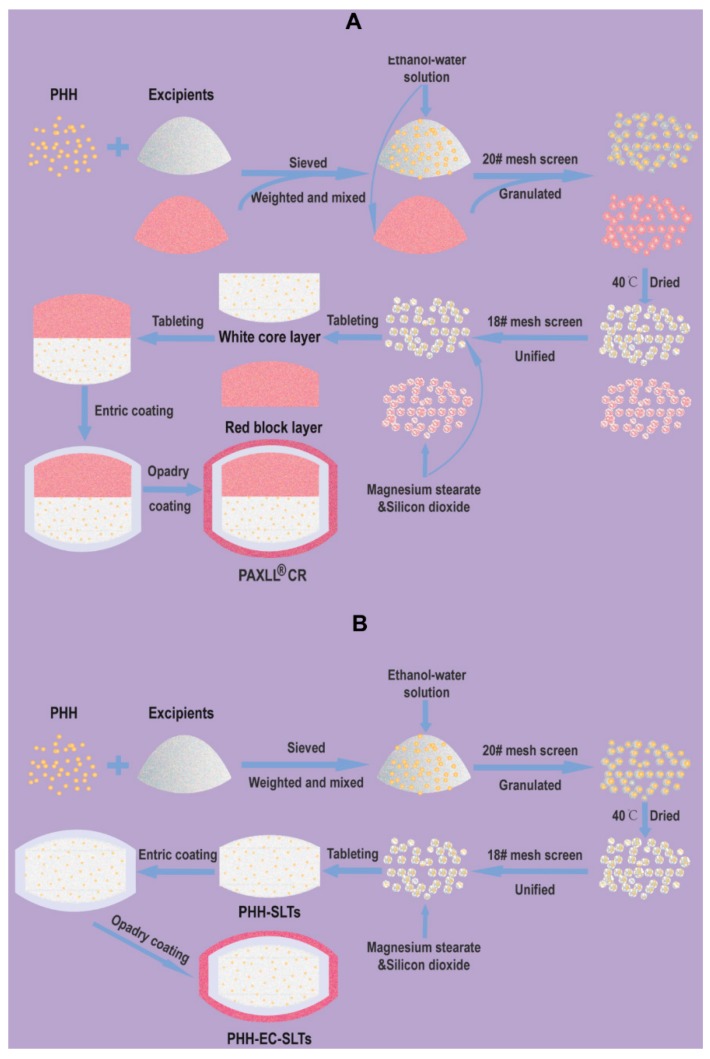
Illustration of PAXIL^®^ CR (**A**) and PHH-EC-SLTs (**B**) preparation procedure.

The formulation for enteric coating of PHH-SLTs was showed in Table 2. Triethyl citrate (TEC) and talcum were weighed, and then 34 g water was added into the mixture. The solution was homogenized by 10,000 rpm high-speed shearing (FA25, Fluko Equipment Shanghai Co., Ltd., Shanghai, China) for 5 min. Then, the homogenate was slowly and steadily poured into the Eudragit L30D-55 aqueous dispersion (30 g polymer with 70 g water), and 40 g water was added in the aqueous dispersion. The mixed solution was subjected to homogenate by 500 rpm stirring for 15 min. A light-proof coating was added additionally with 10% Opadry aqueous dispersion (*w*/*v*). The whole preparation of PHH-EC-SLTs was illustrated by Figure 2.

#### 2.2.4. In Vitro Release Study

Referring to the paddle method described in the general principles 0931 of Chinese Pharmacopoeia IV (2015) and considering the recommended dissolution method about PHH enteric coating controlled-release tablet by FDA, the method of in vitro release investigation was as follows: 

For the PHH-SLTs and PHH double layer controlled-release tablets (PHH-DLTs), they were cast into 1000 mL release medium of pH7.5 Tris-HCl (0.05 mol/L Tris added with 2 mol/L HCl to adjust the pH) and the rotating speed of paddle was 150 rpm. Furthermore, the temperature was controlled at 37.0 ± 0.5 °C. The concentration of PHH in release medium after 0.5, 1.0, 2.0, 4.0 and 6.0 h were measured and then release profiles were acquired. 

In vitro release investigation method of PHH-EC-SLTs and PAXIL^®^ CR was different from above. The dissolution medium was 750 mL of pH 1.0 hydrochloric acid, and then replaced by pH 7.5 Tris-HCl. This was to simulate the circumstance of PHH in the stomach at the first 2 h and in the intestine at the following 6 h. The time intervals were 2.0, 2.5, 3.0, 4.0, 6.0 and 8.0 h.

Besides, filter interference (Table S1) and stability of PHH detected by HPLC (Table S2) were also investigated and shown in Supplementary Material (Section 1). Three batches of PHH-EC-SLTs were prepared and the produce reproducibility was assessed by in vitro release profiles.

#### 2.2.5. Stability of PHH-EC-SLTs

The PHH-EC-SLTs and reference preparation PAXIL^®^ CR were stored under the conditions of high temperature (60 °C), high humidity (RH 75 ± 5%) and strong light irradiation (4500 ± 500 lx), respectively. The appearance, content, related substance and release profiles in vitro after 0, 5 and 10 days storage were investigated.

### 2.3. In Vitro Release Mechanism Study

Following related literatures, specifications and patents [12,13,14,23,24], the PHH-DLTs and coated tablets of PHH-DLTs (PHH-EC-DLTs) were prepared to mimic PAXIL^®^ CR. Relevant data was summarized in Supplementary Material (Section 2). PHH-DLTs were used to replace PAXIL^®^ CR for comparison studies of release mechanism with PHH-SLTs.

#### 2.3.1. Drug Release Models Fitting

Mathematic models would help to interpret the way drug release from the formulations. Four mathematic models including Zero-order, First-order, Higuchi and Korsmeyer-Peppas were fitted with the drug release profiles of PHH-SLTs and PHH-DLTs.

#### 2.3.2. Dynamic Water Absorption and Weight Loss by Erosion in the Drug Release of Tablets

Weight measurement was used to determine the dynamic water intake and weight loss by erosion. More specifically, the initial mass (*w*_0_) of PHH-SLTs and PHH-DLTs were weighed and added into the buffer solution (pH 7.5), the tablets were taken out 0.5, 1.0, 2.0 and 4.0 h later and water was removed carefully. Then, the wet mass and dry mass (drying at 50 °C) of the tablets were weighed. The water content and dry tablet mass were calculated by the Equations (4) and (5), respectively:

(4) $$\mathrm{Water}\;\mathrm{content}\;\left(\%\right)\;\left(t\right)=\frac{\mathrm{wet}\;\mathrm{mass}\;\left(t\right)-\mathrm{dry}\;\mathrm{mass}\;\left(t\right)}{\mathrm{dry}\;\mathrm{mass}\;\left(t\right)}\times 100\%$$

(5) $$\mathrm{Dry}\;\mathrm{tablet}\;\mathrm{mass}\;\left(\%\right)\left(t\right)=\frac{\mathrm{dry}\;\mathrm{mass}\;\left(t\right)}{w_{0}}\;\times 100\%$$

### 2.4. In Vivo Pharmacokinetic Study

#### 2.4.1. Drug Administration

All the procedures and experimental methods carried out in this study were approved by the Institutional Animal Care and Use Committee of Sun Yat-sen University (Guangzhou, China, Approval No.: SYSU-IACUC-2018-000209, 29 October 2018). Four beagle dogs were used for the in vivo pharmacokinetic study of PHH-EC-SLTs and PAXIL^®^ CR by self-contrast method. The beagles were kept in separate cages with free access to water and food. Each dog was dosed by one tablet of PHH-EC-SLTs, and then 4 mL blood samples were collected at 0, 0.5, 1, 2, 3, 4, 6, 8, 10, 12, 24 and 48 h after dosing. With a washout period of two weeks, another tablet of PAXIL^®^ CR was administrated to each dog and blood samples were taken at the same time intervals.

#### 2.4.2. Blood Sampling

Blood samples were stored in an ice bath and centrifuged at 8000 rpm at 4 °C for at least 5 min. The harvested plasma was separated and stored at −20 °C pending analysis. Before the determination of PHH concentration, 0.5 mL methanol was added to 1 mL plasma to precipitate proteins, and the system was subjected to 8000 rpm centrifugation for 5 min. The supernatant was added with 0.1 mol/mL sodium hydroxide solution 0.2 mL to alkalify the solution. Moreover, 3 mL hexyl hydride was used to extract PHH. After 8000 rpm centrifugation for 5 min the upper organic phase was obtained and dried by nitrogen flow. The dried residue was dissolved in 100 µL of 80% methanol water solution (*v*/*v*) and then assayed by HPLC.

#### 2.4.3. Analytical Method

Analysis of PHH concentrations in dog plasma was measured by HPLC analysis with an UltiMate 3000 HPLC system (UV detector VWD-3100, Quaternary Pump SR-3000 and Autosampler LPG-3400SD, Dionex, Waltham, MA, USA). The HPLC analysis conditions were: reversed phase C_18_ analytical column (Gemini C18, 250 × 4.6 mm, 5 µm, Phenomenex, Torrance, CA, USA); mobile phase, 0.07 mol/L ammonium acetate-acetonitrile (70:30, *v*/*v*) which was adjusted the pH value to 5.5 by adding glacial acetic acid. Then, triethylamine was added in to prepare the final mobile phase 0.07 mol/L ammonium acetate-acetonitrile-triethylamine (63:27:10, *v*/*v*/*v*); wavelength, 210 nm; column temperature, 40 °C; flow rate, 1.0 mL/min. Clozapine was used as the internal standard. Calibration was established within the range of 4.02~160.82 ng/mL. And calibration curves were obtained by performing a linear least-squares regression. No interference from endogenous plasma constituents was observed for the analyte or internal standards. The intra-day precision ranged from 0.10% to 3.19% with the accuracy ranging from 0.05% to 0.09%, respectively, and the deviation of inter-assay stability was 2.15%.

## 3. Results and Discussion

### 3.1. Optimal Formulation of PHH-EC-SLTs

The formulation of PHH-EC-SLTs was optimized by factorial design tests and single factor experiments. And the similarity of in vitro dissolution profiles between PHH-EC-SLTs and PAXIL^®^ CR were verified. Producing reproducibility and stability of PHH-EC-SLTs were also explored through comparing the release behaviors in vitro.

#### 3.1.1. Factorial Design Results

It was presumed that the CGS system would achieve zero-order release in this work and HPMC K4M and CMC-Na were chosen to prepare the matrix core of PHH-SLTs. The hydration and strength of these two materials have a great influence on the drug release behavior of tablets made with them. On one hand, the hydration of HPMC K4M was much slower than that of CMC-Na. As a result, single HPMC K4M would have unsatisfactory release resistance capacity at the early stage, while the presence of CMC-Na would help to achieve desired controlled release behavior [25]. On the other hand, the higher gel strength of HPMC K4M compared to CMC-Na could mitigate the destruction of the CGS system. Therefore, the CGS system could avoid the disadvantages of single [26,27,28] and the top priority was to find the optimal content of these two materials.

**Figure 3:**
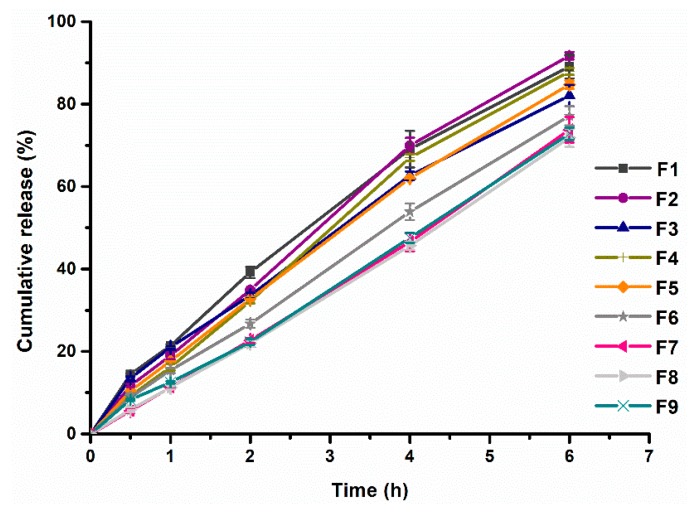
Dissolution profiles of nine formulations from the 3^2^ factorial design. *Y*_60_, *Y*_120_ and *Y*_240_ were the cumulative release percentage at 60, 120 and 240 min, receptivity, *n* = 3.

Factorial design would help to obtain the most appropriate content of HPMC K4M and CMC-Na. Nine formulations (F1~F9) of PHH-SLTs in the 3^2^ factorial design were prepared (Table 1) and the release profiles in vitro were evaluated. The dissolution profiles can be seen in Figure 3. Calculated values of MDT (Equation (1)) and *f*_2_ (Equation (2)) were obtained from the results of dissolution profiles, summarized in Table 3. From F1 to F9 (Table 3), MDT value increased with the increasing content of HPMC K4M. However, the content of CMC-Na had little influence on MDT. 

The variance analysis results (*F*) among nine formulations are shown in Table 4. The *F* value indicated that there were statistically significant differences (*p* < 0.01) among F1 to F9. Both contents of HPMC K4M and CMC-Na had significant influence on the drug release profiles of PHH-SLTs. Also, there was interaction between the content of HPMC K4M and CMC-Na.

To understand the influence of HPMC K4M and CMC-Na on drug release, further binominal regression fitting (Equation (3)) was used. The calculating results were listed in Table 5. One-way ANOVA showed that *Y*_60_, *Y*_120_, *Y*_240_, MDT and *f*_2_ were well fit with Equation (3) (*p* < 0.01), although the fitting degree value of *f*_2_ (*F* = 13.305) was less significant than the other four. As for the absolute value of *b*_1_ and *b*_2_ in *Y*_60_, *Y*_120_, and *Y*_240_, it was found that |*b*_1_| < |*b*_2_| in *Y*_60_ and *Y*_120_ while |*b*_1_| > |*b*_2_| in *Y*_240_. These results suggested that at the beginning of the drug release, the content of CMC-Na had greater influence than HPMC. However, this should be conversely applied to the case 2 h later. These results corresponded with the previous assumption that CMC-Na would compensate for the defects of HPMC K4M which could not maintain a steady drug release at the early stage (0~2 h) because of its much slower hydration rate, but the hydration of CMC-Na would be complete within 2 h, and then HPMC K4M would be the main water-absorbing polymer influencing the drug release and preventing the GCS system from breaking thanks to its high hydration gel strength over the whole release profile. This was why the CGS could avoid the disadvantages of single HPMC K4M and CMC-Na and achieve the desired controlled-release profile. Fully considering the fitting results of MDT and *f*_2_, it is reasonable to state that both HPMC and CMC-Na had a significant influence on the release behavior of the tablets. An appropriate content of HPMC K4M and CMC-Na could avoid their disadvantages and achieve desired controlled-release tablets.

**Figure 4:**
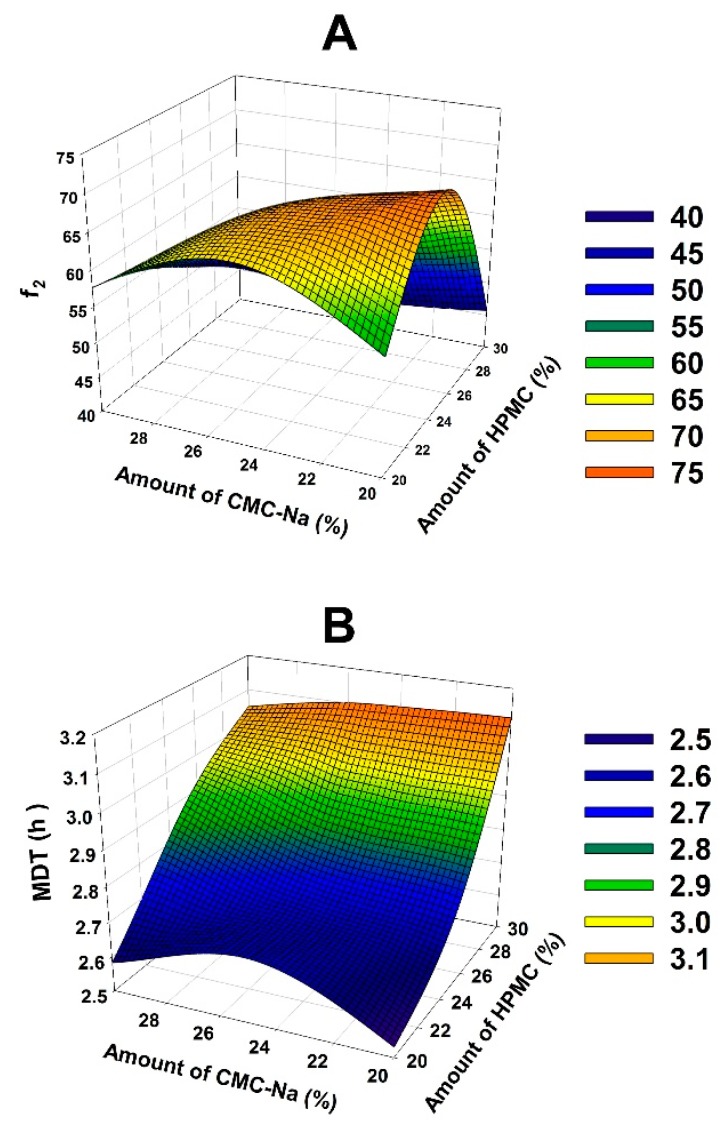
Response surface of *f*_2_ (**A**) and MDT (**B**) as a function of the amount of HPMC and CMC-Na.

The 3D response surface for MDT and *f*_2_ is displayed in Figure 4. They were drawn by Sigma Plot to determine the optimal content of HPMC and CMC-Na. Microsoft Excel 2016 (Microsoft Corporation, Redmond, Washington) was used to solve the regression equation of MDT and *f*_2_. And it was revealed that 23% of HPMC and 24% of CMC-Na should be employed in the optimal formulation of PHH-SLTs. 

#### 3.1.2. Analysis of the Single Factor Experiments Results

**Figure 5:**
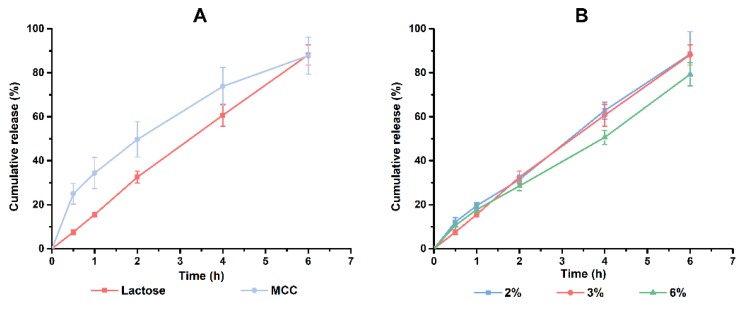
Effect of different types of fillers (**A**) and concentration of adhesives (**B**) on the release of PHH, *n* = 3.

**Figure 6:**
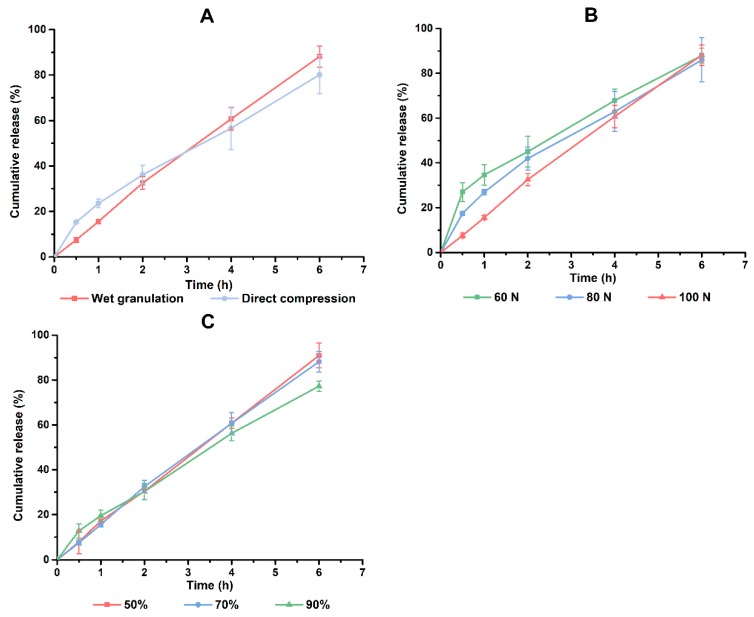
Effect of tableting method (**A**), tableting pressure (**B**) and solvent (**C**) on the release of PHH, *n* = 3.

The content of HPMC and CMC-Na were ascertained based on the factorial design results. Different types of fillers and content of adhesives were also investigated to optimize the formulation of PHH-SLTs. Meanwhile, processing parameters including tableting method, tableting pressure and solvent were determined by single factor experiments. And the related results are shown in Figure 5 and Figure 6.

Lactose and MCC are frequently used fillers in the preparation of tablets. From Figure 5A, faster disintegration of MCC led to markedly faster drug release at the beginning, which was unfavorable for a controlled-release tablet. Thus, lactose was chosen to be the filler. Moreover, the group of 6% PVP K30 would perform a slower release behavior at this period than that of 2% or 3% PVP K30. However, there was no significant difference between these two groups of lower content of PVP K30 (Figure 5B). Taken together, 3% PVP K30 was the better choice. So far, the formulation of PHH-SLTs was determined and displayed in Table 6.

The tablets prepared by a direct compression method would have a faster drug release in the early stage (Figure 6A). Unlike wet granulation where the drug is wrapped in the particles, drugs in direct compression tablets are dispersed as tiny powders and thus produced a faster dissolution rate [29]. In the meantime, the higher porosity of direct compression tablets made the dissolution medium permeate into the tablets more quickly [29]. This accelerated the drug release. Compression pressure has an effect on the porosity of tablets. In general, a lower pressure means a low porosity. This could explain why the burst release ranked in the sequence of 60 N > 80 N > 100 N (Figure 6B). Water and ethanol are the most common solvents used in wet granulation, and an ethanol-water system which avoids the high viscosity of the soft material might be a promising choice. During the tests, it was found that the soft material of 50% and 70% ethanol groups were loose in structure. On the contrary, the 90% group was sticky and smooth. It was supposed that excessive ethanol would inhibit the stretching of the molecular chains of the CGS system. In summary, wet granulation, 100 N of compression pressure and 50% of ethanol concentration were used in the preparation of PHH-SLTs.

#### 3.1.3. In Vitro Release and Producing Process Reproducibility of PHH-EC-SLTs

**Figure 7:**
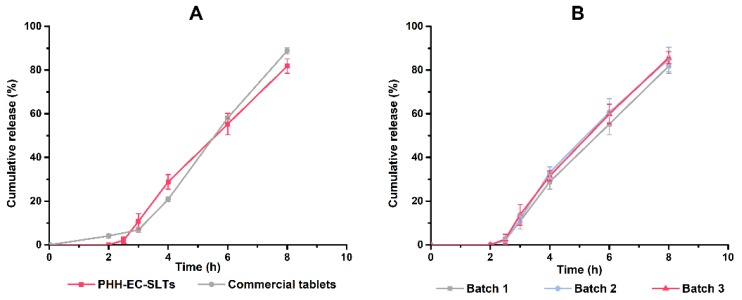
Dissolution profiles of commercial tablets PAXIL^®^ CR and PHH-EC-SLTs (**A**) and produce reproducibility of PHH-EC-SLTs (**B**), *n* = 3.

The in vitro release profiles of optimal formulation of PHH-EC-SLTs showed *f*_2_ = 62.49 > 50 when compared with the commercial formulation. This suggested that PHH-EC-SLTs had similar release behavior with PAXIL^®^ CR (Figure 7A). Moreover, dissolution profile of PHH-EC-SLTs in pH 6.8 (Figure S2A) and 7.2 (Figure S2B) buffer solutions were also measured. And the results provided more evidence that PHH-EC-SLTs were similar to PAXIL^®^ CR in release behavior. The similar release behavior of three batches (*f*_2_ > 50) indicated the production of PHH-EC-SLTs were credible and reproducible (Figure 7B).

#### 3.1.4. Stability of PHH-EC-SLTs

The release profiles in vitro after 0, 5, 10 days storage of PHH-EC-SLTs and PAXIL^®^ CR at three different accelerated stability experiment conditions are displayed in the Supplementary Material (Section 4). Overall, no apparent changes happened in appearance, content, related substance and release behavior of PHH-EC-SLTs. However, there were slightly hygroscopic expansion and delayed drug release (Figure S3B) after 5 and 10 days of exposure to 75 ± 5% humidity. This was because the water molecules inserted in the chain segment of coating polymer as a plasticizer, which rendered the coating film more ductile [30,31]. In addition, when PHH-EC-SLTs were exposed to high temperature (60 °C) and strong light (4500 ± 500 lx), minor delayed release (Figure S3A,B) could occur. Therefore, PHH-EC-SLTs should be sealed under cool and dry conditions.

### 3.2. Release Mechanism of PHH-EC-SLTs

Although there was similar dissolution profile between PHH-EC-SLTs and PAXIL^®^ CR, the certain release mechanisms of them were unclear. A further study was necessary to illuminate their release mechanisms. Therefore, drug release models fitting, dynamic water absorption and erosion weight loss were analyzed to give an explanation in detail.

#### 3.2.1. Analysis of Drug Release Models Fitting

In this work, PHH-DLTs (instead of the commercial tablet) were used when comparing their release profile in vitro while the other parameters (e.g., dissolution profile, stability, pharmacokinetic, bioavailability) were tested by comparing PHH-EC-SLTs and the commercial tablets. Because the PHH-EC-SLTs and PAXIL^®^ CR had the same enteric coating, we would like to study the differences of their release profile detailly without coating. However, it was unscientific to remove the coating off, so we chose to prepare the uncoating core PHH-DLTs of PAXIL^®^ CR to compare with PHH-SLTs. And at the same time, the experiments would be simplified with less disturbances without 2 h release in pH 1.0 hydrochloric acid. In the other experiments, we used PAXIL^®^ CR in order to give a better explanation about the similarity between PHH-EC-SLTs and commercial tablets.

The self-made PHH-DLTs and PHH-EC-DLTs were prepared referring to related literatures, specifications and patents. PAXIL^®^ CR utilized the patented Geomatrix^®^ technology. Geomatrix ^®^ is a technology for oral drug delivery system characterized by multilayer hydrogel matrix tablets [11,12]. Geomatrix^®^ is applicable to obtain multiphase and zero-order release profiles [32]. The results showed that PHH-EC-DLTs (our imitation of PAXIL^®^ CR) had almost exactly the same drug release profile as PAXIL^®^ CR (Figure S1). This meant that using PHH-DLTs as a substitute for the PAXIL^®^ CR core to study release mechanisms was acceptable and credible. Then, mathematical models were constructed for the drug release profile of PHH-SLTs and PHH-DLTs, and the fitting results are listed in Table 7. Dissolution profiles fit better with models when the coefficient *R*^2^ was closer to 1. Thus, both PHH-SLTs and PHH-DLTs were best fit to zero-order release model, whose corresponding *R*^2^ values were 0.9958 and 0.9993, respectively, higher than the other two models. This meant the release profiles in vitro of PHH-SLTs and PHH-DLTs complied with the zero-order equation.

The Korsmeyer-Peppas equation (Equation (6)) could be used to describe drug release from polymeric systems which was the so-called power law [33]:

(6) $$\frac{M_{t}}{M_{\infty }}=kt^{n}$$

where *M*_t_ was the dissolution of drug during a time of *t*, *M*_∞_ was the dissolution amount of drug during zero to infinite time, *k* was the constant related to own structural and geometric characteristics of controlled release system, and *n* was the release exponent, indicative of the mechanism of drug release. Different release exponents, drug release mechanisms [34] and values for spheres and cylinders are displayed in the Table S5 [29,33,35]. The geometry of these two types of self-made tablets was between cylinders and spheres. Their release exponents were *n* > 1, indicating that the release of drug was mainly by polymer matrix erosion.

#### 3.2.2. Dynamic Water Absorption and Erosion Weight Loss

It was demonstrated that both PHH-EC-SLTs and PAXIL^®^ CR released PHH by polymer matrix erosion. However, the drug release during different periods might be quite different. The hydration and strength of HPMC K4M and CMC-Na had great influence on the drug release behavior of tablets made by the CGS system. Water absorption capacity and erosion weight loss of the CGS system when it was put into the dissolution medium could be its hydration and strength characteristics. Therefore, water absorption and erosion weight loss happening during the entire releasing processes were used to illustrate the hydration and strength changes of the CGS system, respectively. Thus, the release mechanism at a specific period of time would be better explained. 

**Figure 8:**
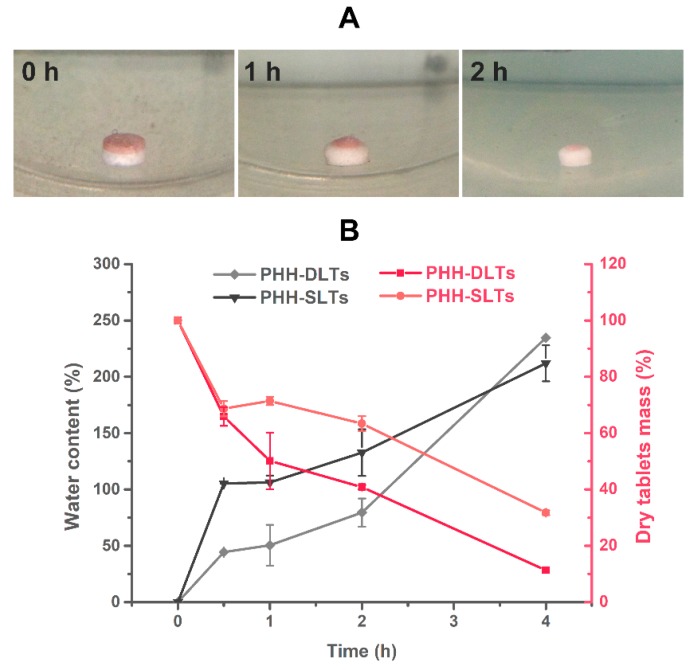
Dynamic water absorption and erosion weight loss experiments, release progress of bilayer tablets from 0 to 2 h (**A**) and water uptake kinetics and changes in the dry mass of PHH-SLTs and PHH-DLTs (**B**), *n* = 3.

The appearance of PHH-DLTs at different release points is shown in Figure 8A. At the same time, the water content and weight loss of PHH-SLTs and PHH-DLTs were obtained from weighing and calculations. The changes of water content and dry tablets mass over time are shown in Figure 8B.

PHH-DLTs had a red block layer with no drug and a white core layer with PHH (Figure 8A). The block layer was eroded faster than core layer during the first 2 h. Considering the changes of water content and weight loss, both PHH-SLTs and PHH-DLTs suffered a large weight loss within the first 0.5 h. The dry tablet masses were 68.68 ± 2.72% and 65.92 ± 3.32%, respectively, but the absorption percentage of PHH-SLTs (105.36 ± 0.14%) was more than twice the rate of PHH-DLTs (44.32 ± 0.11%). This was because the CMC-Na in PHH-SLTs was fairly hydrophilic, while the block layer in PHH-DLTs would protect the core layer from water penetration and slow down its hydration rate. Also, the block layer reduced the surface area from contacting the water, thereby, slowing down the drug release by preventing water penetration and drug diffusion. Nevertheless, the polymer of the block layer has a low viscosity. This made it degrade much faster than the core layer. Because of that, there was a sharp decrease in the dry weight during the early 0.5 h in PHH-DLTs, but in PHH- SLTs, the similar weight loss was mainly due to the swelling of CMC-Na and then lactose dissolving in water. Herein, drug release of both PHH-SLTs and PHH-DLTs were associated with polymer swelling and drug diffusion phenomena.

From 0.5 to 2 h, nearly the same water absorption rate of PHH-SLTs (about 27.45%) and PHH-DLTs (about 35.23%) were observed, although there was drastic difference in their erosion weight loss. These results suggested that the water absorption of CMC-Na reached saturation, and HPMC was the major water-absorbing polymer during this period. The fast erosion of the block layer contributed to the large amount of weight loss of PHH-DLTs. With the swelling of HPMC, the drug release mechanism was a combination of drug diffusion and matrix erosion. For PHH-SLTs, the powerful inter-molecular interaction between swelling HPMC and CMC-Na ensured the strong continuity of the matrix. When the hydrated gel layer was fully formed, the erosion rate of polymers became slower, but the drug diffusion grew faster because of high content of water, and the drug release mechanism was mostly affected by drug diffusion [36]. During the last 2 h, PHH-SLTs and PHH-DLTs absorbed water rapidly again with a high polymer erosion rate, and thus the drug in the tablets showed a rapid and steady release.

### 3.3. In Vivo Pharmacokinetics and Bioavailability

**Figure 9:**
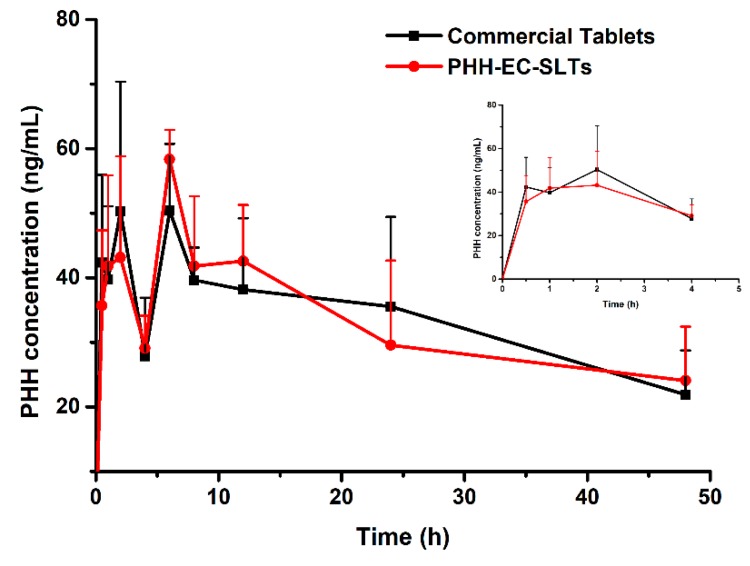
In vivo pharmacokinetic results, PHH concentration in plasma after administration of commercial tablets PAXIL^®^ CR and PHH-EC-SLTs, *n* = 4.

Ultimately, a pharmacokinetics investigation was performed in order to figure out if PHH-EC-SLTs and PAXIL^®^ CR displayed similar in vivo pharmacokinetics profiles. The content of PHH in the plasma of beagles was analyzed at different time points. As shown in Figure 9, the PHH concentration change curves of PHH-EC-SLTs and commercial tablets PAXIL^®^ CR in vivo over time were identical on the whole.

There was enteric coating for both group of tablets. Theoretically, no PHH release would be seen in the first 2 h (gastric acid condition), and this was confirmed by PAXIL^®^ CR pharmacokinetic studies in humans [14,30]. However, PHH was unexpectedly detected after 0.5 h (42.34 ± 13.62 ng/mL for PAXIL^®^ CR and 35.67 ± 11.66 ng/mL for PHH-EC-SLTs). The PHH concentration increased to 50.33 ± 20.05 ng/mL for PAXIL^®^ CR and 43.14 ± 15.69 ng/mL for PHH-EC-SLTs at 2h and then reduced to 27.80 ± 9.08 ng/mL for PAXIL^®^ CR and 29.08 ± 4.99 ng/mL for PHH-EC-SLTs at 4 h. Although no pharmacokinetic studies of PHH enteric controlled-release tablets in beagles were reported, it was well documented that the destructive force in the dog stomach was evaluated to be 3.2 N, which was considerably higher than 1.9 N of humans [37,38]. Hence, it was suggested that the violent peristalsis in gastrointestinal tract of beagles could damage the enteric coating, then PHH is released from collapsed tablets and absorbed in the stomach. Additionally, PHH is a basic drug (pKa was 9.77), meaning that PHH was poorly soluble due to is pH-dependent solubility. It dissolved readily at low pH in the stomach and exhibited a very low solubility at pH values greater than 4. As a result, when the PHH in broken tablets in stomach went in to the intestinal tract, it would be extremely easy to crystallize out, hard and slow to absorb [39,40]. However, the tablets were integrated in vitro, the pH change at 2 h would not lead to PHH separation from the dissolution medium.

The PHH concentrations of both PHH-EC-SLTs and PAXIL^®^ CR in beagles reached the maximum at 6 h, and a slow PHH concentration decline could be observed in the later period. 

The pharmacokinetic parameters of each beagle were processed by WinNonlin 5.01 (Pharsight Corporation, Princeton, NJ, USA) with a non-compartment model. Peak-reaching time (*T*_max_) and peak concentration (*C*_max_) were the tested results and are listed in the Table 8. The log-transformed the area under the blood level-time curve (AUC_0-48_) and *C*_max_ were engaged in variance analysis using SPSS22.0. The analysis results indicated that there was no significant difference between the vital parameters of PHH-EC-SLTs and PAXIL^®^ CR (*p* > 0.05). The relative bioavailability of PHH-EC-SLTs towards PAXIL^®^ CR was very high (*F* = 97.97%). To sum up, there were similar pharmacokinetics between PHH-EC-SLTs and PAXIL^®^ CR in vivo.

PHH-EC-SLTs were prepared based on the CGS system of HPMC and CMC-Na. The 3^2^ factorial design and single factor experiments were used to determine the optimal formulation of PHH-EC-SLTs. PHH-EC-SLTs possessed similar in vitro release behavior with PAXIL^®^ CR. Meanwhile, the preparation process of PHH-EC-SLTs was reproducible, as inferred from the low batch variation. They underwent relatively little change when exposed to the unprotected outside environment, and they could be stored well in a cool and dry place if sealed. The release behavior of PHH-EC-SLTs and PAXIL^®^ CR followed the zero-order equation and they mainly released the drug by polymer matrix erosion. Our release mechanism study showed that CMC-Na hydrated quickly to prevent rapid release of the encapsulated drug during the early stage, and the high gel strength of HPMC K4M could protect the CGS system from deformation. In addition, similar drug release in vivo and oral bioavailability between PHH-EC-SLTs and PAXIL^®^ CR were also confirmed in our in vivo pharmacokinetics study. The results showed that jointly utilizing the different physicochemical property of polymers, hydration capacity and gel strength differences might be an advisable strategy for sustained-release tablet design and development.

## 4. Conclusions

In this work, CGS materials were used to prepare the PHH-EC-SLTs, which imitated the drug release of PAXIL^®^ CR. A 3^2^ factorial design was applied to determine the optimal contents of HPMC and CMC-Na. And PHH-DLTs were used comparatively to study the release mechanism difference with PHH-SLTs. The results showed that there was similar drug release in vivo and oral bioavailability between PHH-EC-SLTs and PAXIL^®^ CR in spite of different drug release mechanisms. In view of simplifying their processing and avoiding risk of double-layer separation, PHH-EC-SLTs could be considered as a prospective alternative of PAXIL^®^ CR, and could be beneficial for advancing the current therapy of MDD.

## Figures and Tables

**Table 1:** Compositions for the 3^2^ factorial design.

Independent Variables	Levels		
	Low	Medium	High
*X*_1_ = content of HPMC K4M (%)	20	25	30
*X*_2_ = content of CMC-Na (%)	20	25	30
Transformed values	−1	0	1

**Table 2:** Formulation of enteric coating for PHH-EC-SLTs.

Ingredient	Amount (g)	Ratio to Polymer (%)
Eudragit L30D-55	100.0	-
TEC	3.0	10.0
Talcum	3.0	10.0
Water	74.0 (evaporated in the end)	-
Solid content	20.0%	
Polymer content	16.7%	

**Table 3:** Responses of the 3^2^ factorial design.

Batch	Variables		Response Values				
	*X*_1_	*X*_2_	*Y*_60_ (%)	*Y*_120_ (%)	*Y*_240_ (%)	MDT (h)	*f*_2_
F1	−1	−1	21.40	39.20	64.67	2.5	56.29
F2	−1	0	18.99	34.89	64.90	2.7	64.58
F3	−1	1	21.02	33.56	59.02	2.6	57.63
F4	0	−1	16.25	32.26	62.04	2.7	70.32
F5	0	0	17.17	32.48	59.84	2.7	66.54
F6	0	1	15.89	28.28	54.13	2.8	55.56
F7	1	−1	12.93	24.53	50.33	3.1	49.59
F8	1	0	11.16	21.86	46.71	3.1	43.80
F9	1	1	12.26	22.48	47.91	3.1	44.97
RE	-	-	13.38	30.26	52.63	3.0	-

RE = reference preparation.

**Table 4:** Analysis of variance for the 3^2^ factorial design of PHH-SLTs.

Parameter	Item	df	MS	*F*
	Model	9	9566.597	1112.504 **
*X*_1_	HPMC K4M	2	796.987	92.682 **
*X*_2_	CMC-Na	2	92.661	10.776 **
*X*_1_ *X*_2_	HPMC CMC	4	102.269	11.893 **
	Error	18	8.599	
	Total	27		

** = *p* < 0.01.

**Table 5:** Binominal regression fitting results of the 3^2^ factorial design.

Responses	Intercept	*b*_1_	*b*_2_	*b*_11_	*b*_22_	*b*_12_	*R* ^2^	*F*
*Y*_60_	47.333	0.556	1.057	0.014	0.013	0.016	0.965	56.135 **
*Y*_120_	60.333	0.197	0.822	0.057	0.017	0.052	0.965	56.135 **
*Y*_240_	46.189	2.276	0.733	0.105	0.053	0.057	0.956	44.568 **
MDT	1.758	0.078	0.105	0.003	0.001	0.001	0.949	38.217 **
*f*_2_	−233.279	19.79	6.033	0.417	0.124	0.015	0.872	13.305 **

** = *p* < 0.01.

**Table 6:** Formulation of PHH-SLTs.

Ingredients	mg/tablet
PHH	25.00
HPMC K4M	36.00
CMC-Na	34.50
Lactose monohydrate	44.25
PVP K30	0.225
Magnesium stearate	1.50
Silicon dioxide	0.75

**Table 7:** Release models and corresponding *R*^2^ values for PHH-SLTs and PHH-DLTs.

Model	Equations	Correlation (*R^2^*)	
		PHH-SLTs	PHH-DLTs
Zero-order	*Q* = 14.364 *t −* 3.016	0.9958	0.9993
First-order	Ln (100- *Q)* = -0.2805 *t* + 4.7388	0.9614	0.8941
Higuchi	*Q* = 35.063 *t*^1/2^ − 14.441	0.9030	0.9046
Korsmeyer-Peppas	*Q* = 12.623 *t*^1.05^	0.9933	0.9994

*Q* = cumulative release percentage, *t* = release time, *R^2^*= correlation coefficient.

**Table 8:** Pharmacokinetics parameters after oral administration in Beagle dogs (*n* = 4).

Pharmacokinetic Parameter	Unit	Values	
		PAXIL^®^ CR	PHH-EC-SLTs
*T*_max_	h	6	6
*C*_max_	ng/mL	50.37 ± 9.14	58.33 ± 10.55
AUC_0-48_	ng·h/mL	1607.86 ± 254.78	1575.24 ± 170.73
AUC_0-∞_	ng·h/mL	2961.65 ± 604.58	3199.52 ± 441.19
*t*_1/2_	h	42.95	46.79
MRT	h	61.59 ± 37.25	68.90 ± 34.72
*V*_d_ */F*	mg·mL/ng	0.53 ± 0.33	0.49 ± 0.13

## Supplementary Materials

The following are available online at http://www.mdpi.com/1999-4923/10/4/243/s1, Figure S1. Dissolution profiles of self-made PHH-EC-DLTS and commercial tablets PAXIL^®^ CR (*n* = 3), Figure S2. Dissolution profiles of PHH-EC-SLTS and commercial tablets PAXIL^®^ CR in different buffers, (A) pH 6.8 buffer solution and (B) pH 7.2 buffer solution (*n* = 3), Figure S3. Dissolution profiles of PHH-EC-SLTs exposed to extreme condition, (A) high temperature (60 °C), (B) high humidity (RH = 75 ± 5%), and (C) strong light (4500 ± 500 lx), *n* = 3, Table S1. Results of filter interference, Table S2. Stability of PHH detected by HPLC, Table S3. Formulation of bilayer tablets, Table S4. Formulation of coating solution for bilayer tablets, Table S5. Exponents (*n*) of Korsmeyer-Peppas model and drug release mechanism from polymeric controlled delivery systems of different geometries.
