# Supplementary material for: Development of Paroxetine Hydrochloride Single Layer Controlled-Release Tablets Based on 32 Factorial Design
Source: Pharmaceutics. 2018 Nov 20;10(4):243. doi: 10.3390/pharmaceutics10040243 (PMC6321141; doi:10.3390/pharmaceutics10040243)
Supplement: Supplementary file 1 [file pharmaceutics-10-00243-s001.zip › pharmaceutics-356209-supplementary.docx]

**Supplementary Material: Development of Paroxetine Hydrochloride Single Layer Controlled-release Tablets Based on 3^2^ Factorial Design**

Yao Yang, Zhengwei Huang, Xuan Zhang, Jiyuan Li, Ying Huang, Wanxin Chen, Xin Pan and Chuanbin Wu

1. Filter Interference and Stability of PHH Detected by HPLC

The ground fine powder of PHH-EC-SLTs was dissolved and diluted with release media to give a solution containing PHH 30 µg/mL. Then a 0.45 µm microporous membrane was used to filter the the solution and the PHH content of the filtrate with the first 1 mL, 3 mL and 5 mL discarded were measured, respectively. As a result, the RSD value was lower than 2% (Table S1), meaning that filter membrane had no influence on the measurements and the PHH solution with 1 mL discarded was measured in the subsequent experiments.

**Table S1.** Results of filter interference.

| Volume of Discarded Primary Filtrate (mL) | 1 | 3 | 5 |
| --- | --- | --- | --- |
| Area (µV⋅s) | 407262 | 395496 | 401959 |
| RSD (%) | 1.67 | | |

The sample of PHH-EC-SLTs was processed with the same method as the filter interference. And the PHH concentration of sample after 0, 4, 8, 12 and 24 h storage were measured by HPLC. It was seen that the peak area of PHH in the release media solution showed no apparent changes with RSD＜2%, and this meant that PHH was relatively stable in the media.

**Table S2.** Stability of PHH detected by HPLC.

| Time (h) | 0 | 4 | 8 | 12 | 24 | RSD (%) |
| --- | --- | --- | --- | --- | --- | --- |
| Area (µV⋅s) | 240266 | 245164 | 239459 | 244294 | 241729 | 1.02 |

2. Formulation of PHH-DLTs and Their Coating Solution

**Table S3.** Formulation of bilayer tablets.

| Core Layer | mg/tablet | Support Layer | mg/tablet |
| --- | --- | --- | --- |
| PHH | 25.00 | HPMC E5 | 30.00 |
| HPMC K4M | 18.75 | Compritol 888 | 15.00 |
| Lactose | 79.14 | Lactose | 29.88 |
| PVP K30 | 2.50 | PVP K30 | 4.00 |
| Magnesium stearate | 1.25 | Magnesium stearate | 0.80 |
| Silicon dioxide | 0.50 | Silicon dioxide | 0.32 |

**Table S4.** Formulation of coating solution for bilayer tablets.

| Coating Solution | mg/tablet |
| --- | --- |
| Eudragit L30D-55 | 41.70 |
| TEC | 1.25 |
| Talcum | 3.12 |
| Water | 16.42 (evaporate in the end) |





**Figure S1.** Dissolution profiles of self-made PHH-EC-DLTS and commercial tablets PAXIL^®^ CR (*n* = 3).

From Figure S1, comparing with PAXIL^®^ CR, it showed *f*_2_ > 50, indicating that the self-made PHH-EC-DTLs had a similar drug release behavior.

3. Dissolution Profiles of PHH in pH 6.8 and pH7.2 Buffer Solution


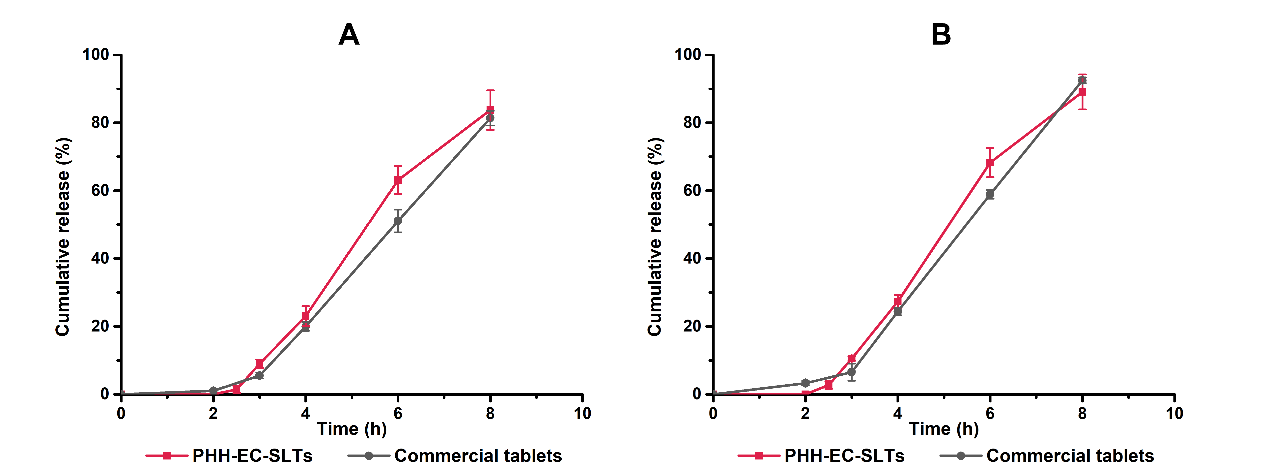


**Figure S2.** Dissolution profiles of PHH-EC-SLTS and commercial tablets PAXIL^®^ CR in different buffers, **(A)** pH 6.8 buffer solution and **(B)** pH 7.2 buffer solution (*n* = 3).

4. Stability Data of PHH-EC-SLTs

| 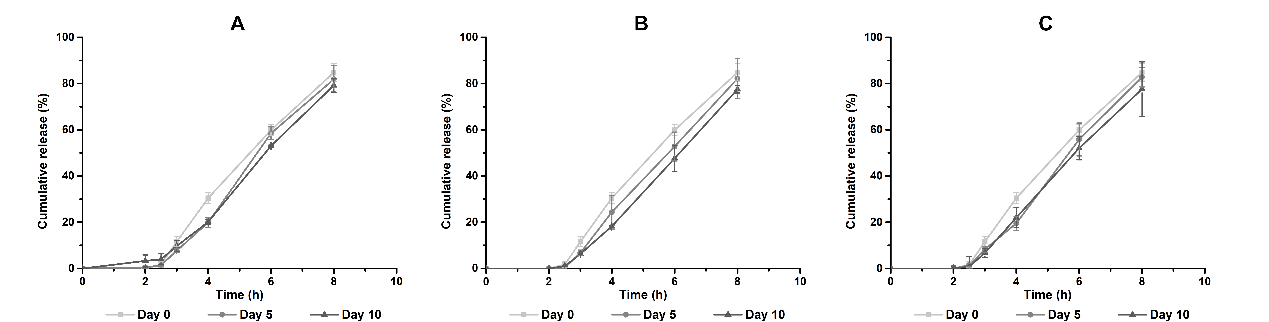 | 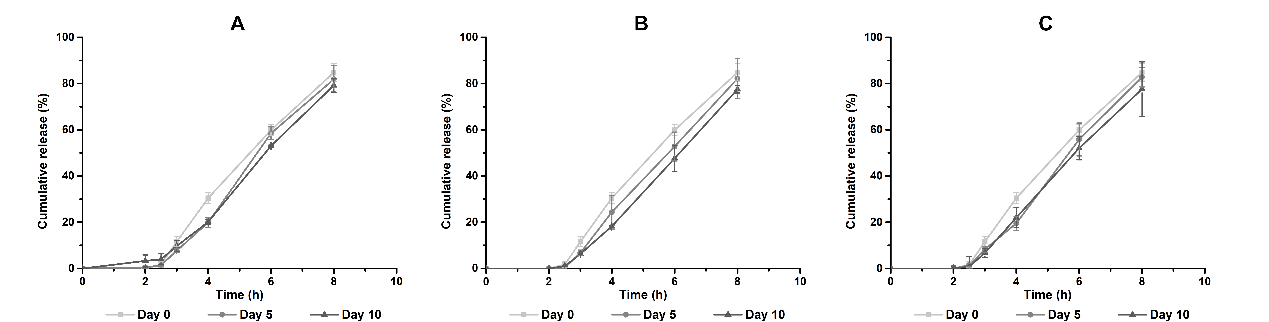 |
| --- | --- |
| 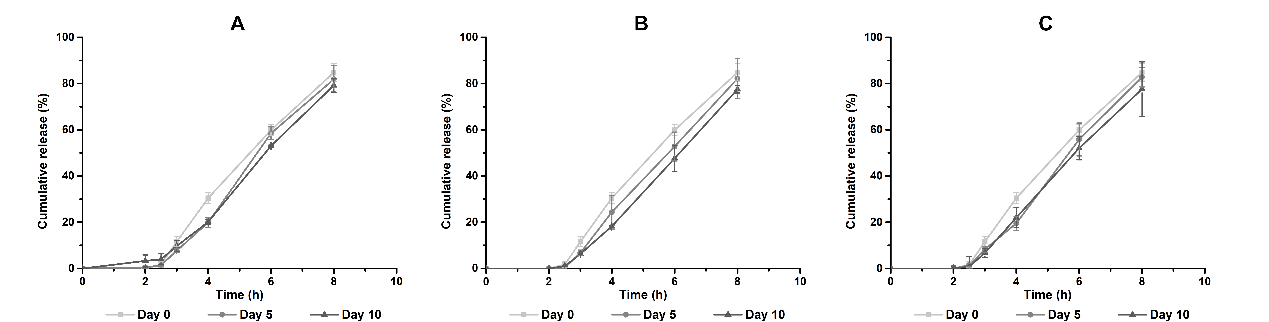 |  |

**Figure S3.** Dissolution profiles of PHH-EC-SLTs exposed to extreme condition, **(A)** high temperature (60℃), **(B)** high humidity (RH = 75 ± 5%), and **(C)** strong light (4500 ± 500 l×), *n* = 3.

5. The Release Index *n* of Korsmeyer-Peppas Model and corresponding Release Mechanism

**Table S5.** Exponents (*n*) of Korsmeyer-Peppas model and drug release mechanism from polymeric controlled delivery systems of different geometries.

| Exponent, *n* | | | Drug Release  Mechanism | Rate as a Function  of Time |
| --- | --- | --- | --- | --- |
| **Thin Film** | **Cylinder** | **Sphere** |  |  |
| 0.50 | 0.45 | 0.43 | Fickian diffusion | $t^{-n}$ |
| 0.50 < *n* < 1.00 | 0.45 < *n* < 0.89 | 0.43 < *n* < 0.85 | Anomalous transport | $t^{n-1}$ |
| 1.00 | 0.89 | 0.85 | Case-II transport | Zero order release |

The terminology of Fickian diffusion, anomalous transport, Case-II transport are used to describe absorption that is linear with the square root of time, has a slight delay or S-shape with the square root of time, or linear with linear time as the table listed equation, respectively, in the column ‘Rate as a function of time’. Thus, in a thin film, it has two distinct physical realistic meanings in the two special cases of *n* = 0.5 (indicating diffusion-controlled drug release) and *n* = 1.0 (indicating swelling-controlled drug release). Values of *n* between 0.5 and 1.0 can be regarded as an indicator for the superposition of both phenomena (anomalous transport). It should be kept in mind that the two extreme values for the exponent *n*, 0.5 and 1.0, are only valid for slab geometry. For spheres and cylinders different values have been derived, as listed in Table S5.
